# De novo ulcerative colitis after kidney transplantation treated with infliximab

**DOI:** 10.1007/s13730-021-00599-6

**Published:** 2021-04-07

**Authors:** Rikako Oki, Sumi Hidaka, Akiko Sasaki, Shinichi Teshima, Yasuhiro Mochida, Katsunori Miyake, Kunihiro Ishioka, Hidekazu Moriya, Takayasu Ohtake, Shuzo Kobayashi

**Affiliations:** 1 Kidney Disease and Transplant Center, Shonan Kamakura General Hospital, 1370-1 Okamoto, Kamakura, Kanagawa 247-0072 Japan; 2 Division of Nephrology and Endocrinology, The University of Tokyo, Tokyo, Japan; 3 Shonan Gastroenterology Medicine Center, Shonan Kamakura General Hospital, Kamakura, Japan; 4 Department of Pathology, Shonan Kamakura General Hospital, Kamakura, Japan

**Keywords:** Kidney transplantation, De novo inflammatory bowel disease, Ulcerative colitis, Diarrhea, Infliximab

## Abstract

Diarrhea is a common complication in kidney transplant recipients. Common causes of diarrhea include infection, side effect from medication, rejection, and malignancy. A less common but important cause of diarrhea is de novo inflammatory bowel disease (IBD). This is unexpected, as these patients are already immunosuppressed. Herein, we present the case of a 45-year-old man with end-stage kidney disease because of focal segmental glomerulosclerosis who underwent preemptive kidney transplantation, with his mother as donor. His immunosuppressive regimen included methylprednisolone, mycophenolate mofetil, and tacrolimus. He had no episodes of graft dysfunction, rejection, or infectious events. Two and a half years post-transplantation, he developed bloody diarrhea. After excluding infections, colonoscopy was performed and revealed edematous mucosa and erythema with pigmentation, which are typical findings in ulcerative colitis. Despite therapy with 5-aminosalicylate and granulocyte monocyte apheresis, he presented with massive bloody diarrhea. We initiated infliximab, an anti-tumor necrosis factor-α (TNF-α) agent. He responded very well and achieved remission within 6 months after initiation of infliximab, while administration of the other immunosuppressants was maintained. His course was uneventful and no complications developed. Management of immunosuppressants for de novo IBD after organ transplantation is complicated, because treatment of IBD, graft function protection, and prevention of infection must be considered. Therefore, cooperation between transplantation physicians and gastroenterologists is essential during therapy.

## Introduction

Widespread use of immunosuppressive agents has dramatically improved patient and organ survival rates after kidney transplantation [1]. With the increase in number of kidney transplantations (KT), various complications have emerged in clinical practice that must be managed and investigated. Gastrointestinal (GI) adverse events are common after KT, with incidence of 20–50% in KT recipients [2]. Diarrhea is a common GI adverse event with various degrees of severity, from mild to fatal. Diarrhea can affect quality of life and drug absorption, and cause great physical burden to patients. Causes of diarrhea include infection, side effect from medication, rejection, and malignancy [2]. Although less common than other causes, de novo inflammatory bowel disease (IBD) was found to be an important cause of diarrhea [2]. Despite already being immunosuppressed, several recent reports demonstrated patients who developed de novo IBD following KT [2, 3]. However, the mechanism, treatment, and management of de novo IBD after solid organ transplantation remain under evaluation, with no standard criteria. An anti-tumor necrosis factor-α (TNF-α) therapy is known to one of options for a corticosteroid-dependent course of UC [4]. To date, there are only limited clinical experience regarding the use of anti TNF-α therapy in UC in KT recipients. Herein, we present the case of a 45-year-old man who developed refractory new-onset ulcerative colitis (UC) under various immunosuppressive agents, 2.5 years after KT. He did not respond to 5-aminosalicylate, standard therapy for UC and had successfully achieved clinical remission with infliximab, an anti TNF-α therapy.

## Case report

A 45-year-old man with end-stage kidney disease because of focal segmental glomerulosclerosis successfully underwent preemptive kidney transplantation 5 years ago, with his mother as donor. His serum creatinine level remained within 1.3–1.4 mg/dL, and no urinary abnormalities were detected while taking tacrolimus (4.0 mg), methylprednisolone (2.0 mg), and mycophenolate mofetil (MMF, 750 mg). Trough level of tacrolimus was monitored once per month, and maintained at less than 5.0 ng/ml from 3 months after KT. Prior to the current presentation, pre-transplantation colonoscopy did not reveal evidence of malignancy or IBD. Following KT, he experienced no complications such as graft dysfunction, rejection episodes, or infectious events.

**Fig. 1:**
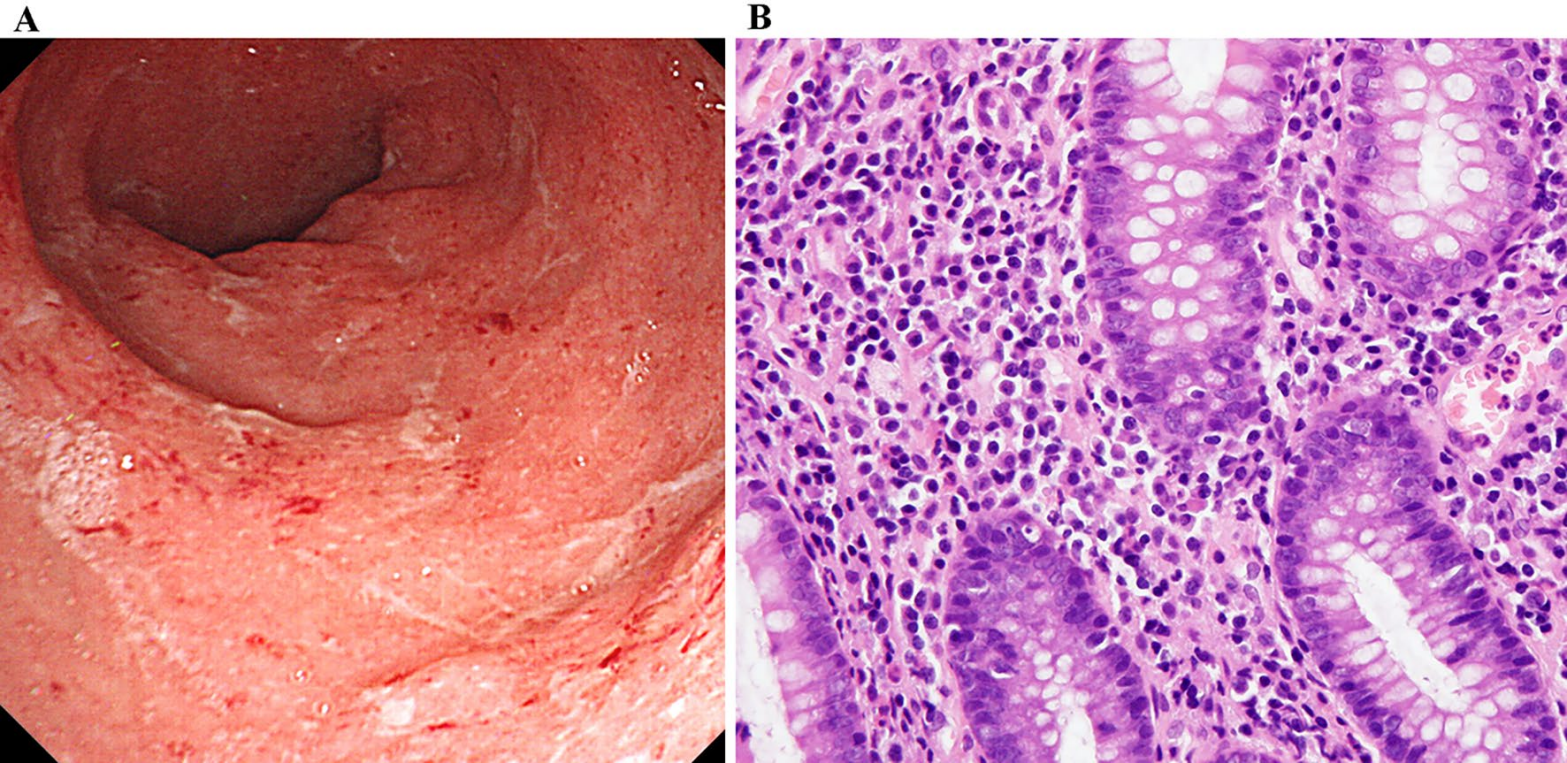
Initial colonoscopy and colon biopsy findings. **a** Colonoscopy revealed edematous mucosa and erythema with pigmentation. **b** Light microscopic examination of colonic mucosa revealed severe inflammation with decreased goblet cells, and crypt abscesses, diffuse stromal lymphoplasmacytic infiltration, and irregular glands, which are consistent with UC (hematoxylin and eosin staining × 200)

**Fig. 2:**
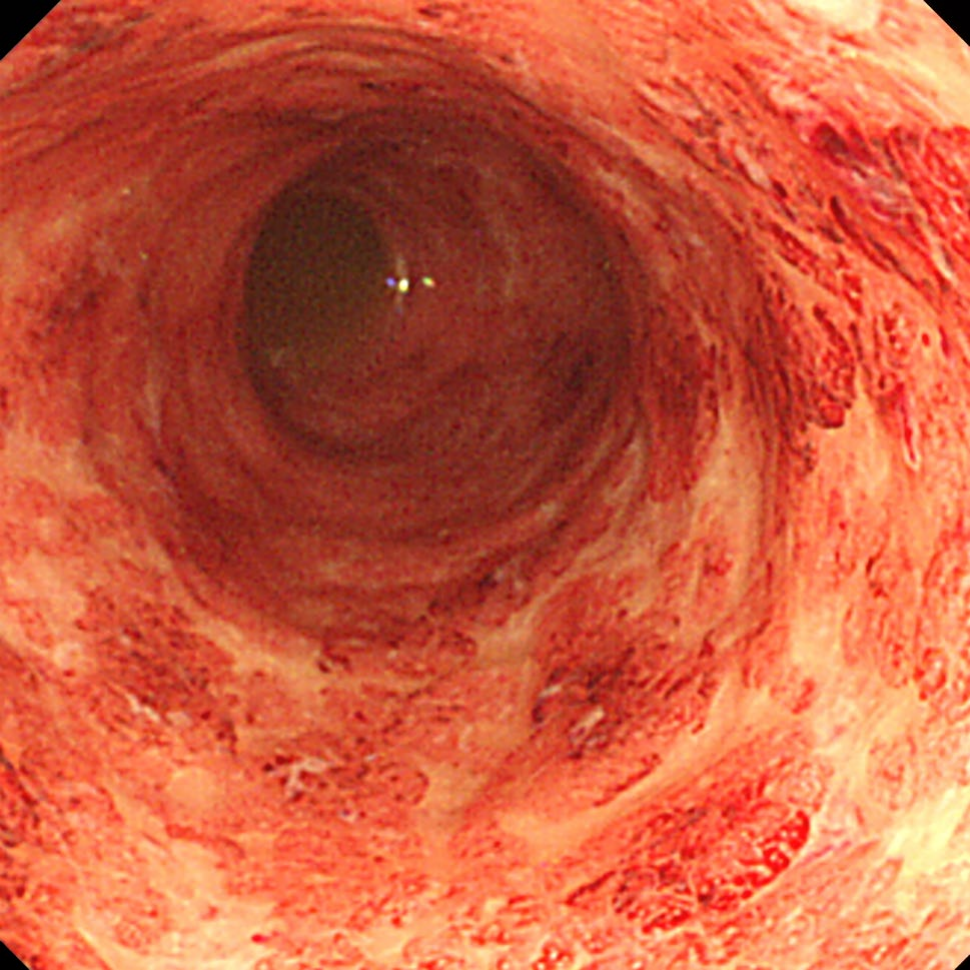
Colonoscopy finding 8 months after starting treatment with 5-ASA and apheresis. Colonoscopy showed mucosal erosion and spontaneous bleeding

**Fig. 3:**
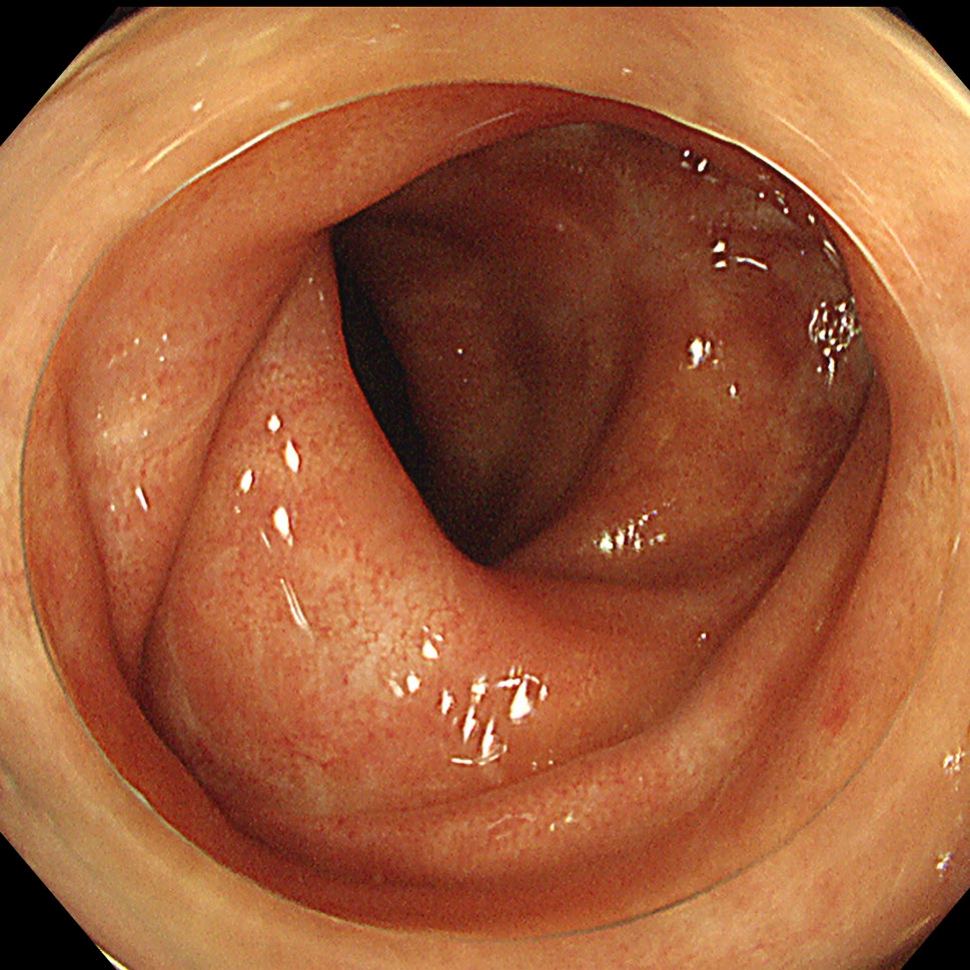
Follow-up colonoscopy 2 years after starting treatment with infliximab. Colonoscopy showed significant improvement of inflammation

Two and a half years post-transplantation, he exhibited diarrhea with bloody stool over 10 times per day. Laboratory findings upon initial presentation of diarrhea did not reveal elevated white blood cell count (4.9 × 10^3^/μl) or serum C-reactive protein (CRP, 0.1 mg/dL) (normal range, < 0.3 mg/dL). His serum creatinine level was 1.36 mg/dL without abnormal urinary findings, which was consistent with the baseline level. Hemoglobin and platelet count were normal (12.0 g/dL and 260 × 10^3^/μl, respectively). Stool cultures were negative for routine bacteriological examination (*Shigella, Escherichia coli,* and *Campylobacter*). Stool specimens were also negative for *Clostridium difficile* toxin. Cytomegalovirus (CMV) infection was ruled out by histological examination of colonic biopsy specimens and negative result from antigenemia assay. Colonoscopy revealed edematous mucosa and erythema with pigmentation, which are typical findings in UC (Fig. 1a). Light microscopic examination of colonic mucosa samples revealed severe inflammation with decreased goblet cells, and crypt abscesses, diffuse stromal lymphoplasmacytic infiltration, and irregular glands, which are also consistent with UC (Fig. 1b). Regarding histological findings, an IBD-like pattern can be observed in MMF colitis [5]. Considering most cases of MMF colitis develop within the first 6 months after onset of treatment [5], in our patient, diarrhea was believed to have resulted from UC, rather than as a side effect of MMF. Therefore, he was started on 3,000 mg of 5-aminosalicylate (5-ASA) and underwent 10 sessions of granulocyte monocyte apheresis. However, he presented with massive bloody diarrhea and serum CRP levels elevated to 2.8–3.0 mg/dL 8 months after starting treatment with 5-ASA and apheresis. Follow-up colonoscopy showed significant deterioration, as there was mucosal erosion and spontaneous bleeding, which were not observed on prior colonoscopy (Fig. 2). CMV infection was excluded by histopathological examination of specimens obtained from colon biopsy. Therefore, he was administered infliximab, anti TNF-α therapy (5.0 mg/kg). Considering the risk of infection from use of multiple immunosuppressants, administration of methylprednisolone was suspended for 2 months and later resumed. He responded very well to the new regimen and achieved remission 6 months after initiation of infliximab. Infliximab has been administered every other month on a continuing basis. Outpatient management of UC has been carefully continued with uneventful clinical course. Follow-up colonoscopy 2 years after starting treatment with infliximab showed significant improvement, based on there being no mucosal erosion or spontaneous bleeding (Fig. 3).

## Discussion

We report the detailed clinical course of a case of de novo UC after KT in the setting of multiple immunosuppressants. The patient achieved remission following initiation of anti TNF-α therapy (infliximab) without any complications, while the doses of other immunosuppressants were maintained. This case supports that anti TNF-α therapy has a potential to be safe treatment option in refractory de novo UC in KT patients.

Although diarrhea is well recognized as a frequent complication following KT, there are no formal guidelines or criteria for its treatment [6]. The Diarrhea Diagnosis Aid and Clinical Treatment (DIDACT) study recommended stopping nonimmunosuppressive drugs associated with diarrhea, and performing microbiological stool examination and viral screening prior to adopting an immunosuppressive regimen [6]. Excluding infectious diseases is an important first step for diagnosis of diarrhea. In a study of 7,103 cases of post-transplant diarrhea, the 3-year cumulative incidence of diarrhea was 22%, over 80% of which were diagnosed with noninfectious diarrhea with unspecified cause [7]. Differentiating MMF-related colitis, CMV infection and post-transplant IBD is particularly challenging because of similar clinical and histological findings. Table 1 showed the endoscopic and histopathological characteristics of above differential diagnosis. The definitive diagnosis of CMV-colitis requires formalin-fixed tissue with immunochemistry and tissue polymerase chain reaction (PCR) [8]. MMF-related colitis is suspected by endoscopic and histological features shown in Table 1, and confirmed when improved only by discontinuation of MMF or a 50% reduction in the initial dose of MMF [8]. It seems essential to judge comprehensively for post-KT diarrhea by clinical symptoms, endoscopic findings, histopathological examination and therapeutic diagnosis. New onset of UC accounted for 13% of all incidences of diarrhea, and was associated with increased risk of graft failure (hazard ratio: 1.31, confidence interval: 1.13–1.52, *p* < 0.05) [7]. According to another report, the incidence of de novo IBD after solid organ transplantation is 10 times higher than that in the overall population (20/100,000 patient-years vs. 206/100,000 patient-years) [9]. Table 2 demonstrated the previously published cases of de novo IBD after kidney transplantation.

**Table1:** Clinical and histological characteristics of diarrhea in KT recipients

	Endoscopic findings	Histological findings
MMF related colitis [5, 8]	Erosions, hyperemia, erythema,	Eosinophils and plasma cells infiltrate, withered crypts, apoptotic bodies
CMV infection [8]	Patchy erythema, exudates, microerosions	Enterocyte apoptosis, inclusion bodies
de novo UC [4, 16]	Erythema, loss of normal vascular pattern, granularity, erosions, friability, bleeding, and ulcerations	Chronic active colitis limited to the rectum
De novo Crohn [8]	Ileitis with multiple ulcers	Expansion of the lamina propria, crypt architectural distortion

**Table2:** previously published cases of de novo IBD after kidney transplantation

Author	Age, sex	Original disease of kidney dysfunction	Presentation of IBD	Maintenance immunosuppressant	Rejection	Duration from kt to uc onset	Treatment for uc	Patient outcome
P. Azevedo [17]	52/M	IgA nephropathy	UC	TAC, MMF	None	5 months	Steroid, 5-ASA	Clinical remission
P. Azevedo [17]	42/F	Familial nephropathy	CD	TAC, MMF, steroid	None	5 years	5-ASA, steroid	Clinical remission
P. Azevedo [17]	40/F	Anti-GBM disease	CD	TAC, MMF, steroid	None	10 years	5-ASA, steroid, AZA	Clinical remission
J. Passfall [18]	60/M	Unknown origin	UC	CyA	None	6 years	Sulfasalazine, steroid, 5-ASA	Clinical remission
A. Hibbs [19]	4/M	Good pasture disease	UC	CyA, steroid,AZA	None	4 years	Surgery	Clinical remission
O. Gheith [20]	26/M	IgA nephropathy	UC	CyA, MMF,steroid	None	5 years	Sulfasalazine	Clinical remission
M. Fernandes* [21]	6/M	Mesangial sclerosis	IBD	TAC,MMF,steroid	–	3 years	5-ASA, steroid, AZA	Clinical remission
M. Fernandes^a^ [21]	13/M	Posterior urethral valves	IBD	TAC,MMF,steroid	–	11 years	Steroid, AZA	Clinical remission
R. Riley [22]	36/M	Obstructive uropathy	IBD	CyA,steroid	–	–	Sulfasalazine	Clinical remission
R. Riley [22]	39/F	Polycystic kidney disease	IBD	CyA,steroid	–	–	Steroid	Intermittent flares

*M* male, *F* female, *UC* ulcerative colitis, *CD* Crohn disease, *GBM* glomerular basement membrane, *TAC* tacrolimus, *MMF* Mycophenolate Mofetil, *AZA* azathioprine,* CyA* Cyclosporine *A,5-ASA* 5-aminosalicylic acid

^a^These cases are reported as IBD-like chronic intestinal inflammation

Although the pathogenesis of de novo IBD after solid organ transplantation remains unclear, some potential mechanisms have been suggested. It was suggested that tacrolimus increases the risk of post-transplant IBD [9]. Haagsma et al. reported that IBD-free survival was significantly higher in patients not receiving tacrolimus, compared with those receiving tacrolimus [10]. Tacrolimus suppresses interleukin-2 production, which reduces generation of regulatory T cells, which in turn are necessary for immunological homeostasis in the intestine [11]. Additionally, mice deficient in interleukin-2 were shown to develop IBD [12]. In our case, long-term use of tacrolimus may have triggered de novo UC. Given the risk of rejection, we did not decrease the dose of tacrolimus. It is well known that MMF is associated with GI side effects, with incidence of approximately 45% in patients receiving the drug [5]. However, whether MMF is related to development of de novo IBD remains controversial [9]. Pretransplant IBD was also reported to be an independent predictor associated with IBD after transplantation [9]. This fact was not applicable in our case because the patient had no history of GI complaints and there were no abnormal findings on colonoscopy pretransplantation.

Generally, 5-ASA is the preferred first-line treatment for UC and oral steroids should be considered for patients who do not respond adequately to 5-ASA [4]. Patients with steroid dependent UC should be treated with an either azathioprine, 6-mercaptopurine, TNF-inhibitor or vedolizumab [4]. Regarding treatment for de novo UC after KT, no standard protocol has been established because of complexities arising from combined use of common IBD therapy and antirejection therapy. Considering previously published data of de novo UC cases in Table 2, 5-ASA, steroid or sulfasalazine have implicated in protective control against deterioration in IBD activity. TNF-α antibody (infliximab) is an established option for refractory IBD [4], but application for KT patients is still a matter of debate. Temme et al. reported the first case reports which demonstrated the clinical remission using infliximab for KT recipients with steroid-refractory UC diagnosed before KT [13]. Garrouste et al. reported seven KT recipients with IBD (5 patients with Crohn’s disease and 2 patients with UC) who were treated with anti-TNF-α therapy. According to their report, over 80% of patients achieved clinical responses [14]. Nonetheless, several complications were observed. Two patients developed infections and developed cancer [14]. In one case with Crohn disease, both acute antibody-mediated rejection and acute T-cell-mediated rejection occurred [14]. In our case, clinical remission has been maintained without any complications such as serious infection events, cancer or rejection. For preventing serious infection and rejection, elaborating the use of multiple immunosuppressants could be a value. We decided to suspend administration of methylprednisolone for 2 months at the timing of anti TNF-α therapy initiation. Confirming that no serious infections developed, methylprednisolone was resumed to reduce the risk of rejection. Williams et al. reported that anti-TNFα therapy was not associated with an increased risk of malignancy in patients with IBD [15]. Regulating bowel inflammation by anti TNF-α therapy might rather contribute to lower the risk of colon cancer. Therefore, anti TNF-α therapy with careful attention to prevention of infection and protection of graft function, could be a therapeutic alternative during aggravation of symptoms in a refractory case.

In conclusion, we report the detailed clinical course of a case of new-onset UC after KT treated with anti TNF-α therapy. De novo IBD should be considered a differential diagnosis in patients presenting post-transplantation diarrhea. Comprehensive evaluation with prompt colonoscopy and histopathological examination is recommended. Cooperation between transplantation physicians and gastroenterologists is essential during therapy.
